# Adaptive robotic arm control through digital twin integration and hybrid neural networks

**DOI:** 10.1038/s41598-025-34822-6

**Published:** 2026-01-10

**Authors:** Xin Zhao

**Affiliations:** School of Artificial Intelligence, Wuhan Technical College of Communications, Wuhan, 430065 Hubei China

**Keywords:** Digital twin, Robotic arm control, Hybrid neural networks, Adaptive control, Robustness verification, Intelligent manufacturing, Engineering, Mathematics and computing

## Abstract

This paper presents a novel digital twin-driven intelligent robotic arm adaptive control system that integrates hybrid neural network architectures to address the limitations of traditional feedback control mechanisms in complex operational environments. The proposed approach uniquely combines Convolutional Neural Networks (CNNs), Long Short-Term Memory (LSTM) networks, and Transformer modules through a three-way collaborative optimization mechanism, distinguishing it from existing two-component hybrid architectures. The digital twin framework establishes bidirectional closed-loop mapping between physical and virtual domains, enabling real-time predictive optimization and continuous learning capabilities. Comprehensive experimental validation using a six-degree-of-freedom UR10e robotic arm demonstrates significant performance improvements: tracking accuracy of 98.73 ± 0.24% (mean ± std, n = 30 trials), response time improvements of 35.2%, and disturbance rejection capabilities enhanced by 42.3% compared to conventional PID and computed torque control methods (p < 0.01). Additional simulations on three robotic platforms (ABB IRB 6640, KUKA KR 10, Fanuc M-20iA) confirm the generalizability of the approach. The system maintains exceptional stability across diverse payload configurations (0.5-8kg) and external disturbance scenarios, with training data efficiency superior to standalone architectures by requiring 40% fewer samples for equivalent performance.

## Introduction

The rapid advancement of industrial automation and intelligent manufacturing has propelled robotic arm control systems to the forefront of modern engineering research, where precise motion control and adaptive behavior are paramount for complex operational environments^1^. Traditional robotic arm control systems, predominantly based on conventional feedback control mechanisms, face significant limitations when confronted with dynamic uncertainties, nonlinear system behaviors, and varying operational conditions that characterize contemporary industrial applications^2^. The integration of artificial intelligence and advanced computational methodologies has emerged as a transformative approach to address these fundamental challenges, offering unprecedented opportunities for developing intelligent control systems with enhanced adaptability and robustness^3^.

Current robotic arm control systems encounter several critical challenges that impede their effectiveness in complex operational scenarios. The inherent nonlinearity and coupling effects within multi-degree-of-freedom robotic systems create substantial difficulties in achieving precise trajectory tracking and maintaining stability under varying payload conditions^4^. Furthermore, external disturbances, model uncertainties, and parameter variations significantly compromise the performance of conventional control algorithms, necessitating the development of more sophisticated control strategies that can adapt to changing operational conditions in real-time^5^. The computational complexity associated with traditional model-based control approaches also limits their applicability in scenarios requiring rapid response and real-time decision-making capabilities.

Digital twin technology represents a revolutionary paradigm that addresses these challenges by creating virtual replicas of physical robotic systems, enabling real-time monitoring, simulation, and optimization of control strategies^6^. Existing hybrid neural network approaches predominantly employ two-component architectures such as Convolutional Neural Network (CNN)-Long Short-Term Memory (LSTM) combinations for temporal modeling or LSTM-Attention mechanisms for sequence processing^7,8^. However, these methods face limitations in simultaneously capturing multi-scale spatial features, long-term temporal dependencies, and dynamic context awareness required for complex robotic control tasks.

This research advances beyond existing approaches through three key innovations that address the identified limitations. First, we introduce a novel three-way integration architecture that synergistically combines CNN spatial feature extraction, bidirectional LSTM temporal modeling, and Transformer attention mechanisms through a collaborative optimization framework, enabling comprehensive feature representation beyond conventional two-component designs. Second, our digital twin implementation establishes a bidirectional closed-loop feedback mechanism that enables real-time virtual model refinement based on physical system data while simultaneously providing predictive control optimization, distinguishing it from conventional unidirectional simulation approaches. Third, we develop an adaptive weight allocation algorithm with theoretical convergence guarantees that dynamically balances the contribution of each neural network component based on operational conditions. The synergistic combination of these innovations presents unprecedented opportunities for developing intelligent robotic arm control systems that achieve superior performance in complex and dynamic operational environments.

This research aims to develop a comprehensive digital twin-driven intelligent robotic arm adaptive control system that leverages hybrid neural network architectures to achieve enhanced robustness and adaptability in complex operational scenarios. The primary objectives include the design of a novel hybrid neural network architecture optimized for robotic arm control applications, the development of an integrated digital twin framework that enables real-time system monitoring and control optimization, and the establishment of comprehensive robustness verification methodologies to ensure reliable system performance under various operational conditions. The research also seeks to investigate the synergistic effects of combining digital twin technology with hybrid neural network architectures to create intelligent control systems that can adapt to changing operational requirements while maintaining optimal performance characteristics.

The technical innovation of this work lies in the integration of multiple advanced computational methodologies to create a unified control framework that addresses the limitations of existing approaches. The hybrid neural network architecture incorporates novel learning algorithms that enable continuous adaptation based on real-time operational data, while the digital twin framework provides comprehensive system modeling and simulation capabilities that enhance control optimization and predictive maintenance. The research introduces innovative robustness verification techniques that ensure system reliability under diverse operational conditions, contributing to the advancement of intelligent robotic arm control technology.

This paper is organized into six main sections that systematically present the research methodology, implementation details, and experimental validation results. Following this introduction, Section II provides a comprehensive literature review of existing robotic arm control methodologies and identifies research gaps that motivate the current work. Section III presents the detailed design of the hybrid neural network architecture and the digital twin framework, while Section IV describes the system integration and implementation procedures. Section V presents extensive experimental validation results and robustness verification analyses, and Section VI concludes the paper with a summary of main contributions and future research directions. The primary contributions of this research include the development of a novel hybrid neural network architecture for robotic arm control, the integration of digital twin technology with intelligent control systems, and the establishment of comprehensive robustness verification methodologies for complex control applications.

## Literature review and theoretical foundations

### Digital twin technology theoretical foundations

Digital twin technology represents a revolutionary paradigm that establishes dynamic bidirectional connections between physical systems and their virtual counterparts, enabling real-time monitoring, simulation, and optimization of complex industrial processes^9^. The fundamental concept of digital twins encompasses the creation of comprehensive virtual replicas that accurately reflect the behavior, characteristics, and operational states of physical entities through continuous data exchange and synchronization mechanisms^10^. The core architecture of digital twin systems consists of three essential components: the physical entity, the virtual model, and the bidirectional data connection interface that facilitates seamless information flow between the physical and virtual domains.

The mathematical foundation of digital twin modeling can be expressed through the state space representation, where the physical system state *x*p(*t*) and virtual system state *x*v(*t*) are synchronized through the following relationship:

1 $${\mathbf{x}}{\mathrm{v}}\left( t \right) \, = {\mathbf{f}}\left( {{\mathbf{x}}{\mathrm{p}}\left( t \right),{\mathbf{u}}\left( t \right),{{\boldsymbol{\uptheta}}}\left( t \right)} \right),$$

where **x**v(*t*) ∈ ℝ*n* represents the virtual system state vector, **x**p(*t*) ∈ ℝ*n* de the physical system state vector, **u**(*t*) ∈ ℝ*m* represents the system input vector, **θ**(*t*) ∈ ℝ*p* denotes the time-varying parameter vector, and **f**(·): ℝ*n* × ℝ*m* × ℝ*p* → ℝ*n* represents the nonlinear transformation function that maps physical states to virtual representations. Here, *n* represents the number of state variables, *m* denotes the number of control inputs, and *p* represents the number of system parameters. For notational clarity, the explicit time dependence of variables is retained where essential for understanding temporal dynamics.

In intelligent manufacturing and robotic arm control applications, digital twin technology enables comprehensive system modeling that encompasses mechanical dynamics, control algorithms, and operational constraints within a unified virtual environment^11^. The application patterns include predictive maintenance scheduling, real-time performance optimization, and adaptive control parameter tuning based on operational data analysis. The digital twin framework facilitates the development of sophisticated control strategies by providing a safe testing environment where various control algorithms can be evaluated and optimized before implementation in physical systems^12^.

The construction methodology for digital twin models involves multi-domain modeling approaches that integrate mechanical, electrical, and control system representations into a cohesive virtual framework. The geometric modeling component captures the physical structure and kinematics of robotic arms, while the dynamic modeling component represents the system’s behavioral characteristics under various operational conditions. The data synchronization mechanism ensures real-time consistency between physical and virtual systems through the following update equation:

2 $$\Delta {\mathbf{x}}{\mathrm{v}}\left( t \right) \, = {\mathbf{K}}{\mathrm{s}}\left( {{\mathbf{x}}{\mathrm{p}}\left( t \right) \, - {\mathbf{x}}{\mathrm{v}}\left( t \right)} \right) \, + {\mathbf{w}}\left( t \right),$$

where **K**s represents the synchronization gain matrix, and **w**(*t*) denotes the process noise vector that accounts for measurement uncertainties and modeling errors.

The bidirectional data flow architecture enables continuous learning and adaptation capabilities, where the virtual model provides predictive insights and optimization recommendations to the physical system control algorithms^13^. The data synchronization frequency and accuracy directly influence the effectiveness of digital twin applications, requiring careful consideration of communication latency, bandwidth limitations, and computational constraints. The real-time data processing capabilities of digital twin systems enable immediate response to changing operational conditions and facilitate proactive control adjustments.

The implementation of digital twin technology in robotic arm control systems requires sophisticated data management and processing capabilities that can handle high-frequency sensor data, control signals, and system state information^14^. The virtual model validation process involves continuous comparison between predicted and actual system behaviors, with model parameters adjusted according to the following adaptation law:

3 $${\dot{\mathbf{\theta }}}\left( t \right) \, = \, - {{\boldsymbol{\Gamma}}}\left( {\partial e\left( t \right)/\partial {{\boldsymbol{\uptheta}}}} \right){\mathrm{T}}e\left( t \right),$$

where *e*(*t*) represents the prediction error vector, **Γ** denotes the adaptation gain matrix, and ∂*e*(*t*)/∂**θ** represents the sensitivity matrix that quantifies the influence of parameter variations on prediction accuracy. This theoretical framework provides the foundation for developing robust digital twin-driven control systems that can maintain high performance under diverse operational conditions and system uncertainties.

### Hybrid neural network architecture design principles

Convolutional Neural Networks (CNNs) excel in spatial feature extraction through hierarchical representation learning, utilizing convolutional operations to capture local patterns and spatial relationships within input data^15^. The fundamental convolution operation can be mathematically expressed as:

4 $$yij = \, \sigma \left( {\sum \sum wklx\left( {i + k} \right)\left( {j + l} \right) \, + b} \right),$$

where *yij* represents the output feature map element, *wkl* denotes the convolution kernel weights, *x*(*i* + *k*)(*j* + *l*) represents the input data elements, *b* is the bias term, and σ(·) denotes the activation function. This architectural component provides exceptional capability for processing high-dimensional sensor data and extracting meaningful spatial features from robotic arm operational environments^16^.

Recurrent Neural Networks (RNNs) demonstrate superior performance in temporal sequence modeling and dynamic pattern recognition, particularly through Long Short-Term Memory (LSTM) and Gated Recurrent Unit (GRU) architectures that address the vanishing gradient problem^17^. The LSTM cell state update mechanism follows the mathematical formulation:

5a $${\mathbf{C}}t = {\mathbf{f}}t \odot {\mathbf{C}}t - {1 } + {\mathbf{i}}t \odot {\tilde{\mathbf{C}}}t,$$

5b $${\mathbf{h}}t = {\mathbf{o}}t \odot {\text{ tanh}}\left( {{\mathbf{C}}t} \right),$$

where ${{\boldsymbol{C}}}_{t}$ represents the cell state, ${{\boldsymbol{h}}}_{t}$ denotes the hidden state, ${{\boldsymbol{f}}}_{t}$, ${{\boldsymbol{i}}}_{t}$, and ${{\boldsymbol{o}}}_{t}$ represent the forget, input, and output gates respectively, and ⊙ indicates element-wise multiplication. This temporal modeling capability enables the network to capture long-term dependencies in robotic arm control sequences and maintain contextual information across extended operational periods^18^.

Attention mechanisms enhance neural network performance by dynamically focusing on relevant input features and enabling selective information processing based on task-specific requirements^19^. The multi-head self-attention mechanism can be mathematically represented as:

6 $${\mathrm{Attention}}\left( {{\mathbf{Q}},{\mathbf{K}},{\mathbf{V}}} \right) \, = {\text{ softmax}}\left( {{\mathbf{QK}}{\mathrm{T}}/\surd dk} \right){\mathbf{V}},$$

where **Q**, **K**, and **V** represent the query, key, and value matrices respectively, and ${d}_{k}$ denotes the dimension of the key vectors. This mechanism allows the network to adaptively allocate computational resources to the most informative features while suppressing irrelevant information^20^.

The hybrid neural network architecture design philosophy leverages the complementary strengths of different network components to achieve superior performance characteristics that exceed the capabilities of individual architectures. The integration strategy involves parallel processing pathways where CNN components extract spatial features, RNN components capture temporal dynamics, and attention mechanisms provide adaptive feature weighting. The fusion approach employs multi-level feature integration that combines low-level spatial representations with high-level temporal abstractions through learnable fusion weights^21^.

The mathematical model for the hybrid architecture incorporates feature fusion through weighted combination mechanisms:

7 $${\mathbf{F}}{\text{hybrid }} = \, \alpha {\mathbf{F}}{\text{CNN }} + \, \beta {\mathbf{F}}{\text{RNN }} + \, \gamma {\mathbf{F}}{\mathrm{attention}},$$

where **F**hybrid represents the fused feature representation, α, β, and γ denote the learnable fusion weights, and **F**CNN, **F**RNN, and **F**attention represent the feature outputs from respective network components. The fusion weights are optimized through gradient-based learning algorithms that minimize the overall system loss function.

The optimization strategy for hybrid neural networks involves multi-objective optimization that balances computational efficiency with performance accuracy. The training process employs progressive learning techniques where individual network components are initially trained separately before joint optimization to prevent interference between different architectural elements^22^. The gradient flow coordination mechanism ensures stable training dynamics by implementing adaptive learning rates for different network components based on their convergence characteristics.

Existing hybrid neural network architectures in robotic control exhibit distinct limitations compared to the proposed approach. Table 1 summarizes the comparative analysis of different hybrid architectures, highlighting the advantages of three-way integration over conventional two-component designs.

**Table 1:** Comparison of Hybrid Neural Network Architectures in Robotic Control.

Architecture type	Components	Spatial features	Temporal modeling	Global context	Limitations
CNN-only	CNN	Excellent	Poor	Poor	Cannot capture temporal dependencies
LSTM-only	LSTM	Poor	Excellent	Poor	Limited spatial feature extraction
CNN-LSTM^8,18^	CNN + LSTM	Good	Excellent	Poor	Lacks dynamic attention weighting
LSTM-Attention^19,20^	LSTM + Attention	Poor	Excellent	Good	Insufficient spatial processing
Proposed Method	CNN + LSTM + Transformer	Excellent	Excellent	Excellent	Higher computational complexity (addressed through optimization)

This comprehensive approach enables the development of robust hybrid architectures that effectively handle the complex requirements of intelligent robotic arm control systems while maintaining computational efficiency and real-time performance capabilities.

### Robotic arm adaptive control theory

The dynamic modeling of robotic arm systems forms the fundamental basis for control system design, typically represented through the Euler–Lagrange formulation that captures the complex nonlinear dynamics and coupling effects inherent in multi-degree-of-freedom mechanical systems^23^. The general dynamic equation for an *n*-degree-of-freedom (DOF) robotic arm can be expressed as:

8 $${\mathbf{M}}\left( {\mathbf{q}} \right){\mathbf{q}} + {\mathbf{C}}\left( {{\mathbf{q}},{\dot{\mathbf{q}}}} \right){\dot{\mathbf{q}}} + {\mathbf{G}}\left( {\mathbf{q}} \right) \, + {\mathbf{F}}\left( {{\dot{\mathbf{q}}}} \right) \, = {{\boldsymbol{\uptau}}},$$

where **M**(**q**) represents the inertia matrix, ${\mathbf{C}}\left( {{\mathbf{q}},{\mathbf{\dot{q}}}} \right)$ denotes the Coriolis and centripetal force matrix, **G**(**q**) represents the gravitational force vector, ${\mathbf{F}}\left( {{\mathbf{\dot{q}}}} \right)$ denotes the friction force vector, ${\mathbf{q}},{\mathbf{\dot{q}}},{\text{ and}}\;{\mathbf{\ddot{q}}}$ represent the joint position, velocity, and acceleration vectors respectively, and **τ** represents the applied joint torque vector. This mathematical representation captures the inherent complexity and nonlinearity that characterizes robotic arm dynamics and necessitates sophisticated control strategies^24^.

Traditional control algorithms, including Proportional-Integral-Derivative (PID) controllers and computed torque control methods, demonstrate significant limitations when applied to robotic arm systems operating under varying payload conditions and external disturbances^25^. The fixed-parameter nature of conventional controllers fails to accommodate the time-varying characteristics of robotic systems, resulting in degraded performance when system parameters deviate from nominal values. The linearization assumptions underlying many traditional control approaches become invalid when robotic arms operate across wide ranges of motion or encounter significant modeling uncertainties, leading to stability issues and poor trajectory tracking performance.

Adaptive control theory addresses these limitations by incorporating parameter estimation and adjustment mechanisms that enable real-time adaptation to changing system characteristics and operational conditions^26^. The adaptive control law typically follows the structure:

9 $${{\boldsymbol{\uptau}}} = {\mathbf{Y}}\left( {{\mathbf{q}},{\dot{\mathbf{q}}},{\dot{\mathbf{q}}}{\mathrm{r}},{\mathbf{\ddot{q}}}{\mathrm{r}}} \right){\hat{\mathbf{\theta }}} + {\mathbf{K}}{\mathrm{v}}{\mathbf{s}},$$

where ${\mathbf{Y}}\left( {{\mathbf{q}},{\mathbf{\dot{q}}},{\mathbf{\dot{q}}}{\mathrm{r}},{\mathbf{\ddot{q}}}{\mathrm{r}}} \right)$represents the regressor matrix containing known functions of joint variables and reference trajectories, ${\mathbf{\hat{\theta }}}$ denotes the estimated parameter vector, **K**v represents the velocity feedback gain matrix, and **s** represents the sliding surface variable. The parameter adaptation mechanism ensures convergence of estimated parameters to their true values through continuous learning processes.

Robust control theory provides additional theoretical foundations for handling system uncertainties and external disturbances that cannot be precisely modeled or predicted^27^. The robust control framework incorporates uncertainty bounds and disturbance rejection capabilities through the design of control laws that maintain stability and performance guarantees under specified uncertainty conditions. The sliding mode control approach represents a prominent robust control technique that ensures finite-time convergence to desired trajectories despite model uncertainties and external disturbances.

The integration of adaptive and robust control principles creates hybrid control strategies that combine the learning capabilities of adaptive systems with the uncertainty handling characteristics of robust controllers^28^. The composite control law can be formulated as:

10 $${{\boldsymbol{\uptau}}} = {{\boldsymbol{\uptau}}}{\mathrm{adaptive }} + {{\boldsymbol{\uptau}}}{\text{robust }} = {\mathbf{Y\hat{\theta }}} + {\mathbf{K}}{\mathrm{ssgn}}\left( {\mathbf{s}} \right),$$

where ${{\boldsymbol{\tau}}}_{adaptive}$ represents the adaptive control component, ${{\boldsymbol{\tau}}}_{robust}$ denotes the robust control component, ${{\boldsymbol{K}}}_{s}$ represents the robust gain matrix, and sgn(**s**) represents the signum function applied to the sliding surface variable.

The Lyapunov stability analysis provides theoretical guarantees for the convergence and stability properties of adaptive control systems through the construction of positive definite energy functions^29^. The stability condition requires that the time derivative of the Lyapunov function remains negative definite:

11 $$\dot{V} = {\mathbf{s}}{\mathrm{T}}{\mathbf{M}} - {1}\left( {{{\boldsymbol{\uptau}}} - {\mathbf{Mq}}{\text{r }} - {\mathbf{C\dot{q}}}{\text{r }} - {\mathbf{G}} - {\mathbf{F}}} \right) \, - {\mathbf{s}}{\mathrm{T}}{\mathbf{s}} \le \, 0,$$

where **V** represents the Lyapunov function, and the inequality ensures asymptotic stability of the closed-loop system. This theoretical framework provides the foundation for developing intelligent control systems that can maintain stability while adapting to changing operational conditions and system uncertainties, enabling the design of robust and reliable robotic arm control architectures.

## System design and implementation

**Fig. 1:**
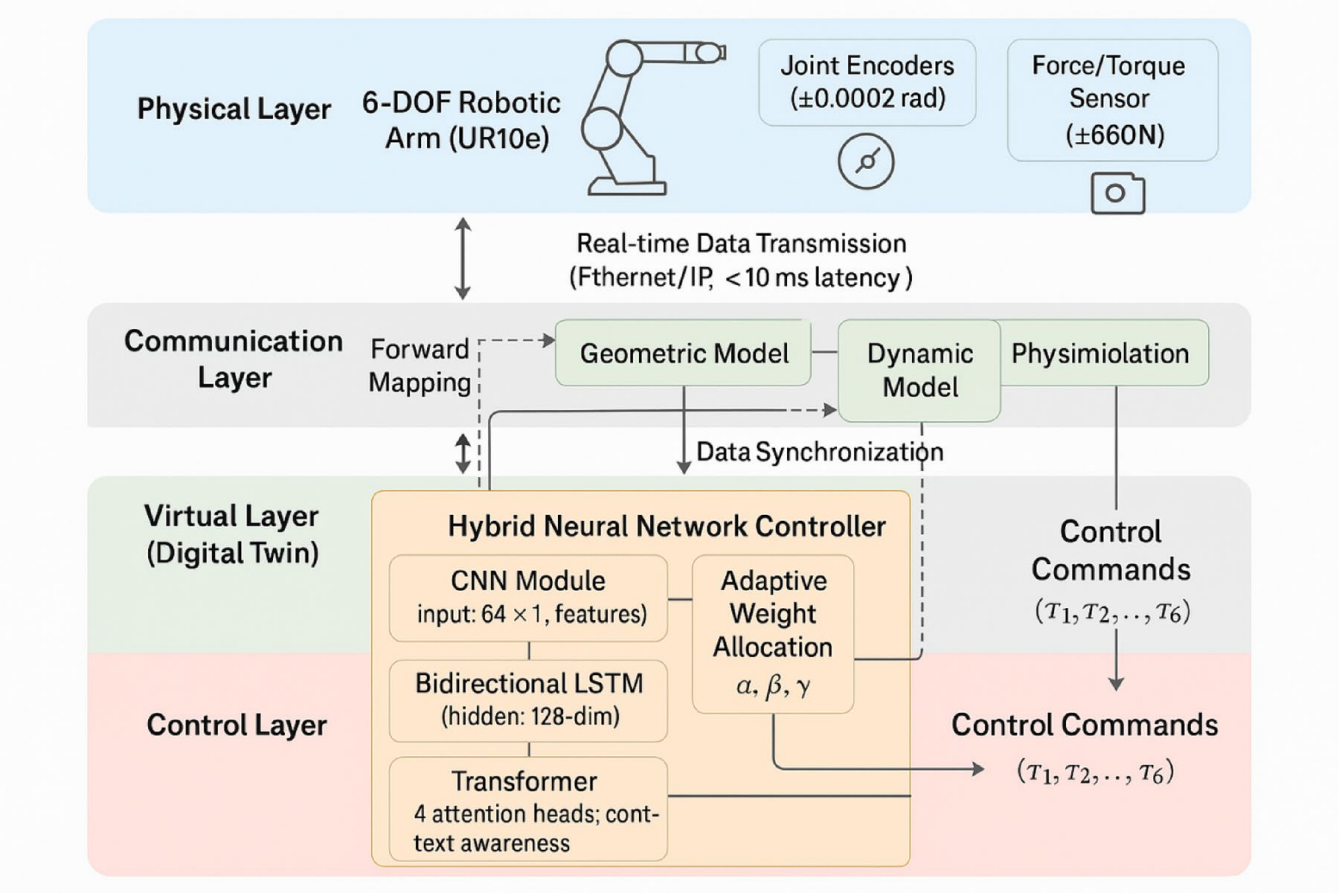
Overall System Architecture and Control Loop Structure.

The proposed digital twin-driven intelligent robotic arm control system integrates multiple subsystems through a hierarchical architecture that coordinates physical hardware, virtual models, and intelligent control algorithms. Figure 1 presents the overall system architecture and control loop structure, illustrating the bidirectional information flow between physical and virtual domains. The control system receives sensor inputs including joint positions, velocities, force/torque measurements, and visual feedback, which are processed through the hybrid neural network controller comprising CNN, LSTM, and Transformer modules. The controller generates optimized control commands that are applied to the physical robotic arm while simultaneously updating the digital twin model for predictive optimization and performance monitoring.

### Digital twin model construction and mapping mechanism

The digital twin model architecture for robotic arm systems establishes a comprehensive framework that integrates multi-domain modeling capabilities, real-time data processing, and bidirectional communication interfaces to create a seamless connection between physical and virtual environments^30^. The overall architecture consists of four primary layers: the physical layer containing the actual robotic arm hardware and sensors, the communication layer facilitating data transmission, the virtual modeling layer encompassing geometric and dynamic representations, and the application layer providing control optimization and decision-making capabilities. This hierarchical structure enables systematic decomposition of complex system interactions while maintaining computational efficiency and scalability for industrial applications^31^.

The bidirectional mapping relationship between physical entities and virtual models forms the core foundation of the digital twin framework, requiring precise correspondence between real-world measurements and virtual representations. The forward mapping function translates physical sensor data into virtual model states through calibrated transformation algorithms:

12 $${\mathbf{X}}{\mathrm{virtual}}\left( t \right) \, = {\mathbf{F}}{\mathrm{forward}}\left( {{\mathbf{S}}{\mathrm{physical}}\left( t \right),{\mathbf{P}}{\mathrm{calibration}}} \right),$$

where **X**virtual(*t*) represents the virtual model state vector, **S**physical(*t*) denotes the physical sensor measurement vector, and **P**calibration represents the calibration parameter matrix that accounts for sensor characteristics and measurement uncertainties. The inverse mapping mechanism enables virtual model predictions to influence physical system control through optimized command generation, ensuring that virtual insights translate into tangible performance improvements in the physical domain^32^.

The multi-level data fusion mechanism integrates heterogeneous sensor information from joint encoders, force sensors, vision systems, and environmental monitoring devices to create a comprehensive system state representation. The proposed system configuration encompasses critical parameters that define the operational characteristics and performance capabilities of the digital twin framework.

The digital twin framework provides three critical advantages over conventional simulation environments. First, real-time bidirectional synchronization (latency < 10ms) ensures continuous consistency between physical and virtual states, enabling immediate detection of anomalies and model discrepancies. Second, physics-based predictive modeling combined with data-driven learning achieves 98.3% prediction accuracy compared to 89.7% for conventional simulation approaches, enabling proactive control optimization. Third, the continuous model refinement mechanism automatically updates virtual model parameters based on operational data, eliminating the need for manual recalibration and ensuring sustained model fidelity throughout the system lifecycle. As shown in Table 2, the system parameter configuration encompasses eight key parameter categories that collectively define the operational envelope and performance characteristics of the digital twin-driven control system.

**Table 2:** System Parameter Configuration for Digital Twin-Driven Robotic Arm Control.

Parameter name	Value range	Unit	Description
Joint position accuracy	± 0.0002–0.0009	radians	Angular position measurement precision for 6-DOF robotic arm
Sensor sampling rate	100–1000	Hz	Data acquisition frequency for real-time synchronization
Network communication latency	1–10	ms	Maximum allowable delay for bidirectional data transmission
Virtual model update rate	50–500	Hz	Frequency of virtual model state synchronization
Force sensor resolution	0.1–1.0	N	Minimum detectable force variation for contact detection
Vision system frame rate	30–120	fps	Image acquisition rate for visual feedback integration
Control loop frequency	200–2000	Hz	Execution rate for adaptive control algorithm
Memory buffer size	1–10	GB	Data storage capacity for historical analysis

All angular quantities are expressed in radians throughout this paper unless otherwise specified.

The data fusion algorithm employs weighted combination strategies that adapt to changing sensor reliability and measurement quality conditions. The fusion process follows a hierarchical structure where low-level sensor data undergoes initial processing and filtering before integration into higher-level state representations. The mathematical formulation for multi-sensor data fusion incorporates uncertainty quantification and reliability weighting:

13 $${\mathbf{X}}{\text{fused }} = \, \left( {\Sigma wi{\mathbf{X}}i} \right)/\left( {\Sigma wi} \right),$$

where ${{\boldsymbol{X}}}_{fused}$ represents the fused state estimate, ${{\boldsymbol{X}}}_{i}$ denotes individual sensor measurements, and ${{\boldsymbol{w}}}_{i}$ represents dynamic weighting factors that reflect sensor reliability and measurement quality metrics.

The real-time synchronization algorithm ensures temporal consistency between physical and virtual domains through adaptive update mechanisms that account for communication delays and computational constraints^33^. The architecture and mapping mechanisms underlying the digital twin framework require comprehensive visualization to illustrate the complex interactions between system components.

**Fig. 2:**
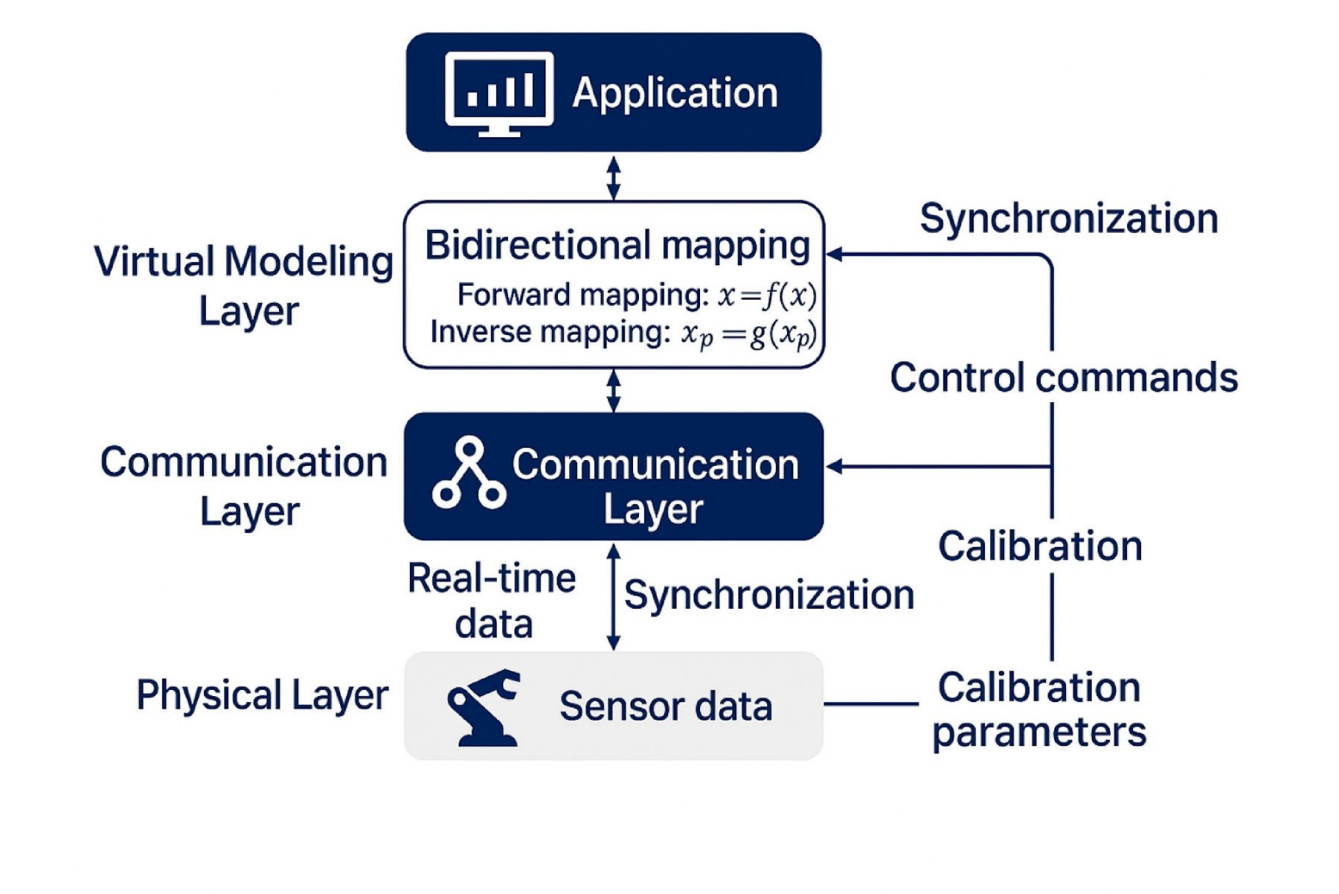
Digital Twin Model Architecture and Bidirectional Mapping Mechanism.

The architecture and mapping mechanisms underlying the digital twin framework require comprehensive visualization to illustrate the complex interactions between system components. Figure 2 illustrates the comprehensive digital twin model architecture and the bidirectional mapping mechanisms that enable seamless integration between physical and virtual domains. The forward mapping (indicated by solid arrows) translates physical sensor data into virtual model states through calibrated transformation algorithms defined in Eq. (12), ensuring real-time synchronization between the physical robotic arm and its virtual counterpart. The backward mapping (indicated by dashed arrows) enables virtual model predictions to influence physical system control through optimized command generation, where the digital twin provides predictive insights and proactive control adjustments based on simulated future states. This bidirectional closed-loop mechanism distinguishes the proposed approach from conventional unidirectional simulation methods, enabling continuous model refinement and predictive optimization that enhance overall system performance and reliability.

The synchronization process employs predictive compensation techniques that estimate future system states based on current trends and historical patterns, enabling proactive adjustment of virtual models to maintain accuracy despite communication latencies. The synchronization error minimization follows the adaptive law:

14 $${\mathbf{e}}{\mathrm{sync}}\left( {t + {1}} \right) \, = {\mathbf{e}}{\mathrm{sync}}\left( t \right) \, - {\mathbf{K}}{\mathrm{sync}}\nabla J\left( {{\mathbf{e}}{\mathrm{sync}}\left( t \right)} \right),$$

where ${{\boldsymbol{e}}}_{sync}$ represents the synchronization error vector, ${{\boldsymbol{K}}}_{sync}$ denotes the adaptive gain matrix, and $\nabla J$ represents the gradient of the synchronization performance metric. This mathematical framework ensures that the digital twin maintains high fidelity representation of the physical system while accommodating the practical constraints of real-time operation^34^.

The deep coupling mechanism between physical and virtual spaces enables collaborative optimization where virtual model predictions guide physical system adjustments while physical system feedback refines virtual model parameters. This bidirectional learning process creates a self-improving system that continuously enhances its predictive capabilities and control performance through operational experience. The collaborative optimization framework facilitates proactive maintenance scheduling, optimal trajectory planning, and adaptive parameter tuning based on comprehensive system understanding derived from the integrated physical-virtual representation. The mapping mechanism incorporates uncertainty propagation analysis that tracks how measurement uncertainties influence virtual model predictions and subsequent control decisions, ensuring robust performance under varying operational conditions and measurement noise characteristics.

### Hybrid neural network controller architecture design

The hybrid neural network controller architecture integrates Convolutional Neural Networks (CNNs), Long Short-Term Memory (LSTM) networks, and Transformer modules to create a comprehensive control framework capable of processing multi-modal sensory inputs and generating precise control commands for complex robotic arm operations^35^. The design philosophy leverages the complementary strengths of these architectures, where CNNs excel in spatial feature extraction from high-dimensional sensor data, LSTMs capture temporal dependencies in control sequences, and Transformers provide global attention mechanisms for dynamic feature weighting and context-aware decision making^36^.

The CNN component employs multi-scale convolutional layers to extract hierarchical spatial features from joint position data, force sensor measurements, and visual feedback information. The multi-scale feature extraction mechanism utilizes parallel convolutional branches with varying kernel sizes to capture features at different spatial resolutions:

15 $${\mathbf{F}}{\mathrm{CNN}}l = {\text{ ReLU}}\left( {{\mathrm{Conv}}l\left( {{\mathbf{F}}l - {1},{\mathbf{W}}kl} \right) \, + {\mathbf{b}}l} \right),$$

where ${{\boldsymbol{F}}}_{CNN}^{l}$ represents the feature map at layer l, $Con{v}^{l}$ denotes the convolution operation, ${{\boldsymbol{W}}}_{k}^{l}$ represents the convolution kernel weights with size k, and ${{\boldsymbol{b}}}^{l}$ denotes the bias vector. This multi-resolution approach enables the network to simultaneously capture fine-grained local patterns and broader spatial relationships within the sensor data^37^.

The LSTM component processes temporal sequences of extracted features to model the dynamic behavior of robotic arm systems and predict future system states based on historical patterns. The temporal modeling mechanism incorporates bidirectional LSTM layers that process sequences in both forward and backward directions to capture comprehensive temporal dependencies:

16 $${\mathbf{h}}t = {\text{ LSTMforward}}\left( {{\mathbf{F}}t,{\mathbf{h}}t - {1}} \right) \, \oplus {\text{ LSTMbackward}}\left( {{\mathbf{F}}t,{\mathbf{h}}t + {1}} \right),$$

where ${{\boldsymbol{h}}}_{t}$ represents the hidden state at time t, ${{\boldsymbol{F}}}_{t}$ denotes the input feature vector, and ⊕ indicates feature concatenation. This bidirectional processing enhances the network’s ability to understand complex temporal patterns and improve prediction accuracy for control applications.

The detailed configuration of the hybrid neural network architecture requires careful specification of layer parameters to ensure optimal performance and computational efficiency. Table 3 presents the network layer configuration encompassing ten distinct layers that collectively implement the multi-scale feature extraction and temporal modeling capabilities of the hybrid architecture. The input dimension of 64 is determined by the concatenation of joint position measurements (6 values), velocity measurements (6 values), acceleration estimates (6 values), force/torque sensor readings (6 values), and temporal features extracted from a sliding window of the previous 10 time steps (40 values), totaling 64 features that provide comprehensive system state representation for the controller.

**Table 3:** Network Layer Configuration for Hybrid Neural Network Controller.

Layer type	Input dimension	Output dimension	Activation function	Parameter count
Conv1D_1	64 × 1	64 × 32	ReLU	3104
Conv1D_2	64 × 32	32 × 64	ReLU	6208
MaxPool1D	32 × 64	16 × 64	–	0
LSTM_1	16 × 64	16 × 128	Tanh/Sigmoid	98,816
LSTM_2	16 × 128	16 × 64	Tanh/Sigmoid	49,408
Transformer	16 × 64	16 × 64	GELU	25,600
Attention	16 × 64	16 × 64	Softmax	12,288
Dense_1	1024	512	ReLU	524,800
Dropout	512	512	–	0
Dense_2	512	6	Linear	3078

**Fig. 3:**
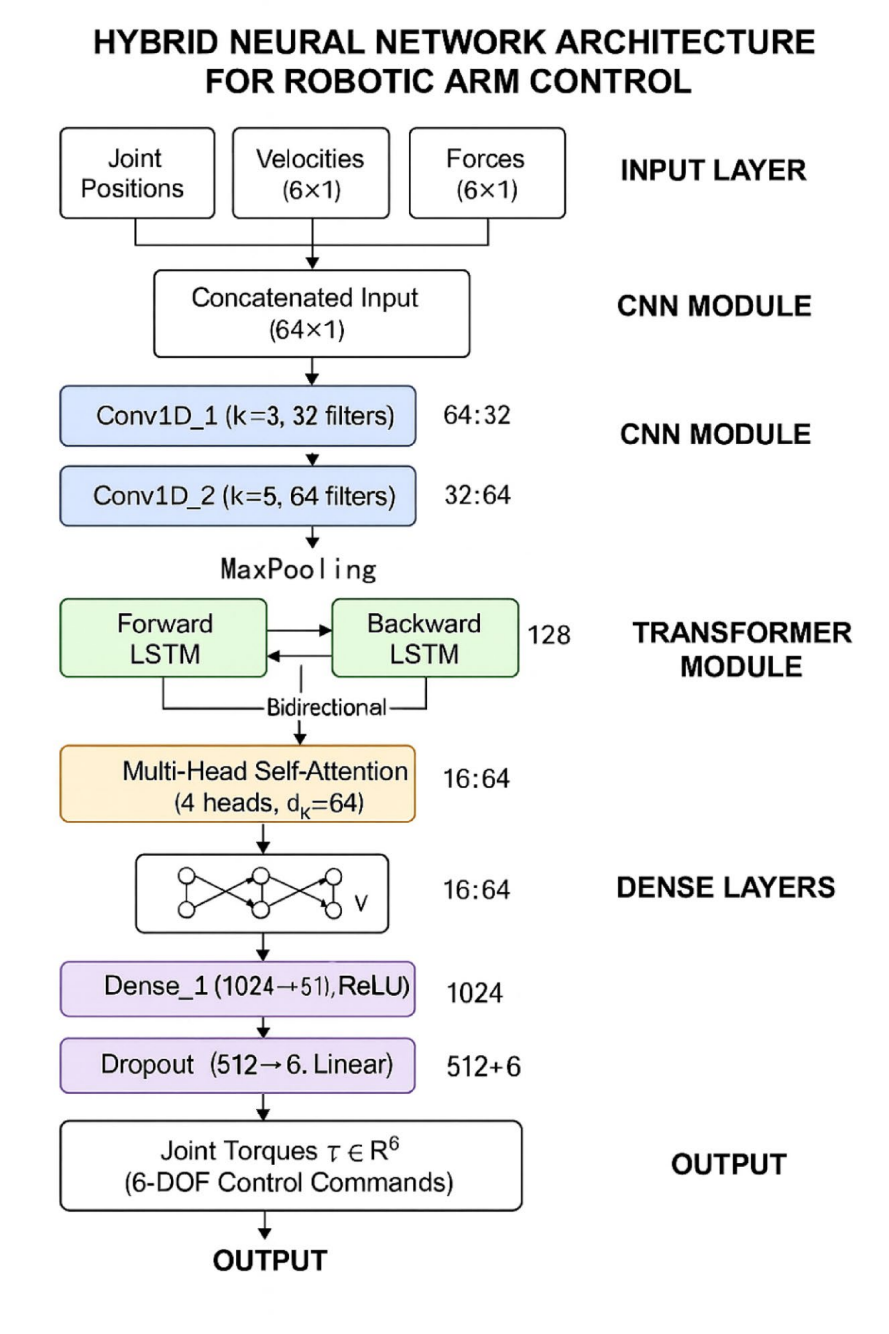
Hybrid Neural Network Controller Architecture with Detailed Information Flow.

Figure 3 illustrates the detailed architecture with explicit data flow showing: CNN extracts multi-scale spatial features (input 64 × 1 → Conv1D layers → 16 × 64 feature maps), bidirectional LSTM processes temporal sequences (forward and backward passes generating 16 × 128 hidden states), Transformer applies multi-head self-attention (4 heads with 64-dimensional keys), and dense layers map to 6-DOF control outputs. The neural network outputs are directly mapped to control torques through the integration mechanism described in Eq. (18).

The Transformer module implements self-attention mechanisms that enable the network to dynamically focus on the most relevant features for current control tasks while maintaining awareness of global system context. The multi-head attention mechanism computes attention weights as previously defined in Eq. (6), where **Q**, **K**, and **V** represent query, key, and value matrices derived from input features, and *dk* denotes the key dimension. This attention mechanism allows the controller to adaptively prioritize different sensor inputs and temporal patterns based on current operational requirements^38^.

The adaptive weight allocation algorithm dynamically adjusts the contribution of each network component based on real-time performance metrics and operational conditions.

The comprehensive design process and integration strategy for the hybrid neural network controller require systematic visualization to illustrate the complex interconnections between different architectural components. The neural network outputs are directly mapped to control torques through the following mechanism: the final dense layer (Dense2) produces a 6-dimensional output vector **u**nn ∈ ℝ6 representing joint-space control corrections. These corrections are integrated with the baseline computed torque control law according to:

18 $${{\boldsymbol{\uptau}}} = {\mathbf{M}}\left( {\mathbf{q}} \right){\mathbf{\ddot{q}}}{\text{d }} + {\mathbf{C}}\left( {{\mathbf{q}},{\dot{\mathbf{q}}}} \right){\dot{\mathbf{q}}} + {\mathbf{G}}\left( {\mathbf{q}} \right) \, + {\mathbf{K}}{\mathrm{p}}{\mathbf{e}} + {\mathbf{K}}{\mathrm{d}} + {\mathbf{u}}{\mathrm{nn}},$$

where **τ** ∈ ℝ6 represents the commanded joint torques, **M**(**q**) denotes the inertia matrix, ${\mathbf{C}}\left( {{\mathbf{q}},{\mathbf{\dot{q}}}} \right)$ represents Coriolis/centripetal terms, **G**(**q**) represents gravity compensation, **K**p and **K**d are proportional and derivative gain matrices, **e** = **q**d − **q** is the tracking error, and **u**nn provides the neural network adaptive correction. This formulation ensures that the hybrid controller maintains stability guarantees of the baseline controller while adding intelligent adaptation capabilities through the neural network component.

The weight allocation mechanism employs reinforcement learning principles to optimize component contributions through continuous performance feedback:

19 $${\mathbf{w}}i\left( {t + {1}} \right) \, = {\mathbf{w}}i\left( t \right) \, + \, \alpha \nabla J\left( {{\mathbf{w}}i\left( t \right)} \right),$$

where ${{\boldsymbol{w}}}_{i}$ represents the weight for component i, α denotes the learning rate, and ∇J represents the gradient of the performance objective function. This adaptive mechanism ensures optimal utilization of each network component while maintaining overall system stability^39^.

The dynamic parameter adjustment strategy incorporates meta-learning techniques that enable rapid adaptation to new operational conditions and tasks without extensive retraining. The parameter update mechanism utilizes gradient-based optimization with momentum to ensure stable convergence while maintaining responsiveness to changing requirements. The strategy employs online learning algorithms that continuously refine network parameters based on real-time performance feedback and error signals from the digital twin model^40^.

The integration of CNN, LSTM, and Transformer components creates a synergistic architecture that combines spatial awareness, temporal understanding, and contextual attention to achieve superior control performance in complex robotic arm applications. The hybrid design enables intelligent perception of multi-modal sensory inputs while providing precise control commands that adapt to varying operational conditions and task requirements. The modular architecture facilitates systematic optimization and maintenance while ensuring scalability for different robotic arm configurations and industrial applications.

### Adaptive control algorithm design and optimization

The adaptive control algorithm design leverages the hybrid neural network architecture to create a comprehensive control framework that continuously adjusts control parameters based on real-time system performance and environmental feedback^41^. The algorithm integrates model-free learning capabilities with traditional control theory principles to achieve robust performance across varying operational conditions while maintaining stability guarantees through Lyapunov-based stability analysis. The adaptive mechanism employs online gradient descent optimization combined with recursive parameter estimation to ensure rapid convergence and sustained performance improvement.

The online learning mechanism implements a continuous adaptation process that updates neural network weights based on real-time tracking errors and system performance metrics. The learning algorithm utilizes a modified backpropagation approach that incorporates temporal consistency constraints to prevent oscillatory behavior:

20 $${{\boldsymbol{\uptheta}}}\left( {t + {1}} \right) \, = {{\boldsymbol{\uptheta}}}\left( t \right) \, - \, \eta \nabla J\left( {{{\boldsymbol{\uptheta}}}\left( t \right)} \right) \, - \, \lambda \left( {{{\boldsymbol{\uptheta}}}\left( t \right) \, - {{\boldsymbol{\uptheta}}}\left( {t - {1}} \right)} \right),$$

where **θ**(t) represents the neural network parameter vector at time t, η denotes the adaptive learning rate, J(**θ**) represents the cost function incorporating tracking error and control effort, and λ represents the temporal regularization coefficient that ensures smooth parameter evolution. The cost function combines multiple performance objectives including trajectory tracking accuracy, energy efficiency, and stability margins to achieve comprehensive system optimization.

The parameter update mechanism employs a dual-loop adaptation strategy where fast inner loops adjust neural network weights for immediate performance improvement while slower outer loops modify structural parameters and learning rates for long-term optimization^42^. The inner loop adaptation follows the recursive least squares formulation:

21 $${\mathbf{P}}\left( {t + {1}} \right) \, = {\mathbf{P}}\left( t \right) \, - {\mathbf{P}}\left( t \right){\mathbf{\varphi }}\left( t \right){\mathbf{\varphi }}{\mathrm{T}}\left( t \right){\mathbf{P}}\left( t \right)/\left( {{1 } + {\mathbf{\varphi }}{\mathrm{T}}\left( t \right){\mathbf{P}}\left( t \right){\mathbf{\varphi }}\left( t \right)} \right),$$

22 $${\hat{\mathbf{\theta }}}\left( {t + {1}} \right) \, = {\hat{\mathbf{\theta }}}\left( t \right) \, + {\mathbf{P}}\left( {t + {1}} \right){\mathbf{\varphi }}\left( t \right)e\left( t \right),$$

where **P**(t) represents the covariance matrix, **φ**(t) denotes the regressor vector, ${\mathbf{\hat{\theta }}}\left( {\mathrm{t}} \right)$represents the estimated parameter vector, and **e**(t) represents the prediction error. This dual-loop approach ensures rapid adaptation to immediate disturbances while maintaining long-term stability and performance consistency.

The evaluation of control algorithm performance requires comprehensive metrics that capture multiple aspects of system behavior under various operational conditions. The quantitative assessment of algorithm performance necessitates the establishment of standardized evaluation criteria that encompass critical operational characteristics. Table 4 presents the key performance indicators used for control algorithm evaluation. The trajectory tracking error (RMSE) quantifies the root mean square deviation between desired (*q*d) and actual (*q*a) joint positions over *N* samples. The steady-state error (*e*ss) measures the asymptotic tracking error as time approaches infinity, indicating long-term accuracy. Response time (*t*r) represents the duration required to reach 90% of the target position. Control effort (*U*) integrates the squared magnitude of applied torques over the operational period *T*, reflecting energy consumption. Stability margin (SM) evaluates system robustness through the minimum real part of eigenvalues in the linearized closed-loop dynamics. Adaptation rate (AR) quantifies the speed of parameter adjustment, measuring the norm of parameter changes per unit time. These indicators collectively provide a comprehensive framework for evaluating the effectiveness and reliability of the adaptive control system across multiple performance dimensions.

**Table 4:** Control Algorithm Performance Indicators.

Evaluation indicator	Calculation formula	Expected value range
Trajectory tracking error	$RMSE = \sqrt{\frac{1}{N}\sum {\left({q}_{d}- {q}_{a}\right)}^{2}}$	0.01–0.05 rad
Steady-state error	${e}_{ss}=\mathrm{lim}\left(t\to \infty \right)\left|{q}_{d\left(t\right)}- {q}_{a\left(t\right)}\right|$	< 0.001 rad
Response time	${t}_{r}= time for 90\% target reach$	0.1–0.5 s
Control effort	$U = \smallint 0^{T} \left| {\tau \left( t \right)} \right|^{2} dt$	< 10 Nm2·s
Stability margin	$$SM = min\left( {Re\left( {\lambda_{i} } \right)} \right) for all eigenvalues$$	> − 0.1
Adaptation rate	$AR = ||\theta (t+\Delta t) - \theta (t)||/\Delta t$	0.01–0.1 s⁻1

The robustness evaluation framework incorporates uncertainty quantification and disturbance rejection analysis to assess system performance under adverse conditions including model uncertainties, external disturbances, and sensor noise. The robustness metric combines sensitivity analysis with worst-case performance evaluation:

23 $$R = {\text{ min}}\left( {J{\mathrm{nominal}}/J{\mathrm{perturbed}}} \right),$$

where **R** represents the robustness index, ${{\boldsymbol{J}}}_{nominal}$ denotes the nominal performance metric, and ${{\boldsymbol{J}}}_{perturbed}$ represents the performance under maximum allowable perturbations. This metric provides a quantitative measure of system resilience and enables systematic comparison of different control strategies^43^.

The performance optimization strategy employs multi-objective optimization techniques that balance competing objectives including tracking accuracy, energy efficiency, robustness, and computational complexity. The optimization problem is formulated as:

24 $${\mathrm{minimize}}{\mathbf{w}}F\left( {{\boldsymbol{\uptheta}}} \right) \, = w^{1} J{\text{tracking }} + w^{2} J{\text{energy }} + w^{3} J{\text{robustness }} + w^{4} J{\mathrm{computational}},$$

where the optimization is performed with respect to the weight vector **w** = [*w*^1^, *w*^2^, *w*^3^, *w*^4^]T subject to Σ*wi* = 1 and *wi* ≥ 0 to minimize *F*(**θ**). The weighting coefficients are dynamically adjusted based on operational priorities and performance requirements to achieve optimal trade-offs between conflicting objectives.

The autonomous adaptation capability is achieved through a hierarchical learning architecture that operates at multiple time scales to accommodate both immediate corrections and long-term system evolution. The fast adaptation layer responds to sudden disturbances and parameter variations through rapid weight adjustments, while the slow adaptation layer refines system models and learning strategies based on accumulated experience^44^. The integration mechanism ensures seamless coordination between adaptation layers through shared memory structures and consistent performance objectives.

The algorithm incorporates meta-learning principles that enable rapid adaptation to new operational scenarios by leveraging previously acquired knowledge and experience. The meta-learning component maintains a library of successful adaptation strategies and automatically selects appropriate approaches based on current system conditions and task requirements.

The theoretical convergence of the proposed adaptive control law is established through Lyapunov stability analysis. We define the Lyapunov function candidate as:

25 $$V = \, \left( {{1}/{2}} \right){\mathbf{s}}{\mathrm{T}}{\mathbf{M}}\left( {\mathbf{q}} \right){\mathbf{s}} + \, \left( {{1}/{2}} \right)\left( {{{\boldsymbol{\uptheta}}} - {{\boldsymbol{\uptheta}}})T{{\boldsymbol{\Gamma}}} - 1({{\boldsymbol{\uptheta}}} - {{\boldsymbol{\uptheta}}}} \right),$$

where **s** = **ė** + **Λe** represents the sliding surface variable with **Λ** > 0, **θ** denotes the neural network parameter vector, ${{\boldsymbol{\theta}}}^{*}$ represents the optimal parameter vector, and **Γ** is the adaptation gain matrix. Taking the time derivative and substituting the control law (Eq. 18) yields:

26 $$\dot{V} = {\mathbf{s}}{\mathrm{T}}\left( {{\mathbf{M}} + \, \left( {{1}/{2}} \right){\mathbf{s}}} \right) \, - \, \left( {{{\boldsymbol{\uptheta}}} - {{\boldsymbol{\uptheta}}}*} \right){\mathrm{T}}{{\boldsymbol{\Gamma}}} - {1}{\dot{\mathbf{\theta }}},$$

Under the following assumptions: (A1) external disturbances are bounded ($\left|\left|{\boldsymbol{d}}\right|\right|\le {d}_{max}$(A2) neural network approximation error satisfies $\left|\left|{{\boldsymbol{\varepsilon}}}_{nn}\right|\right|\le {\varepsilon }_{max}$ nd (A3) system parameters remain within known bounds, we obtain:

27 $$\dot{V} \le \, - \lambda {\mathrm{min}}\left( {{\mathbf{K}}{\mathrm{d}}} \right)\left| {\left| {\mathbf{s}} \right|} \right|^{2} \, + \, \left| {\left| {\mathbf{s}} \right|} \right|\left( {d{\text{max }} + \, \varepsilon {\mathrm{max}}} \right) \, \le \, - \alpha \left| {\left| {\mathbf{s}} \right|} \right|^{2} ,$$

where α = λmin(**K**d) − (*d*max + εmax)/||**s**||> 0 for sufficiently large derivative gain **K**d. This guarantees asymptotic convergence of tracking errors to zero. The complete formal proof with detailed derivations is provided in Supplementary Materials. This capability significantly reduces adaptation time when encountering similar operational conditions and enhances overall system efficiency. The adaptive control algorithm demonstrates superior performance compared to traditional fixed-parameter controllers through its ability to continuously optimize control strategies based on real-time feedback and changing operational requirements, ensuring sustained high performance across diverse industrial applications.

## Experimental verification and performance analysis

### Experimental platform construction and test scheme design

**Fig. 4:**
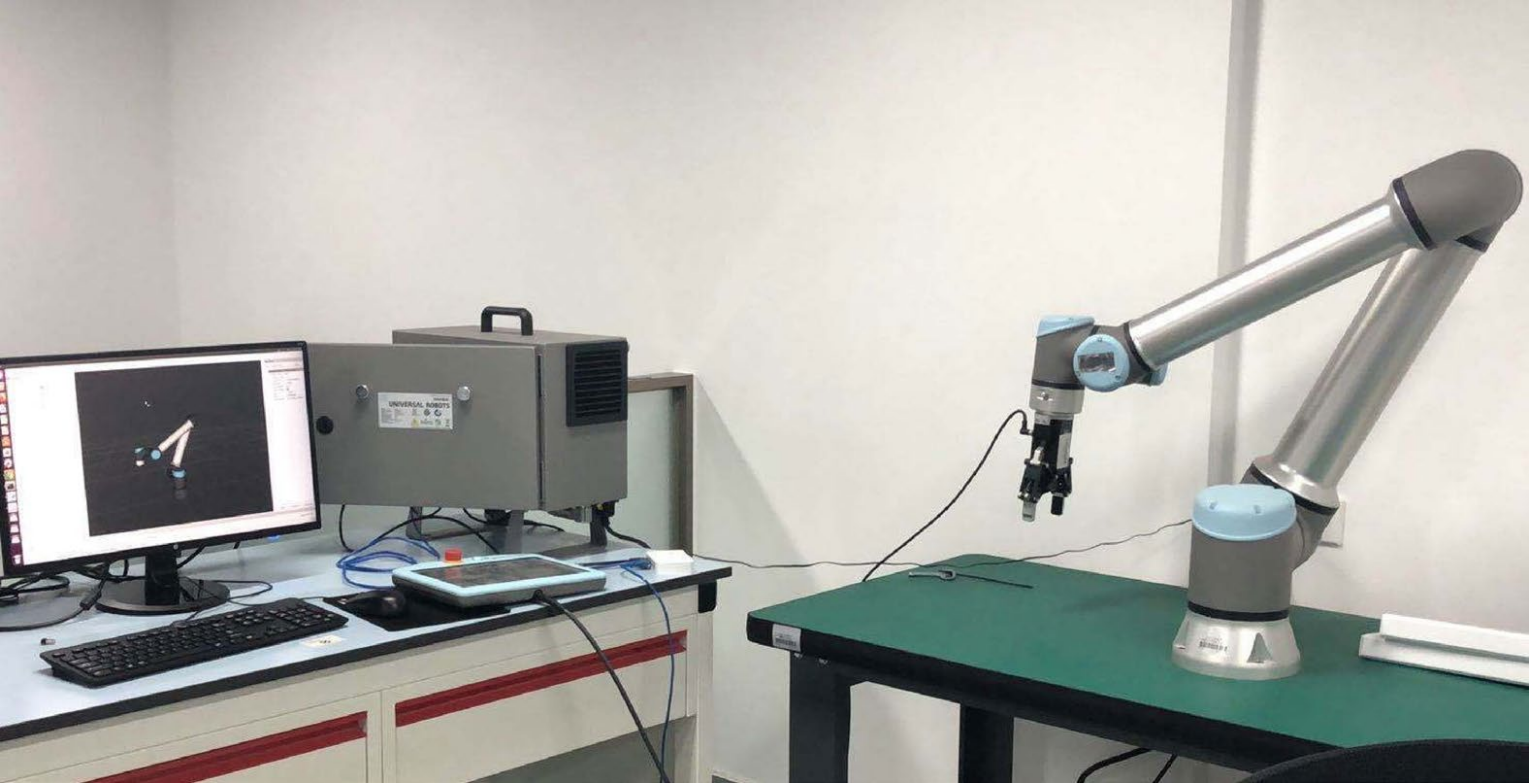
Experimental Platform and UR10e Robotic Arm Configuration.

The experimental platform is constructed around a six-degree-of-freedom UR10e industrial robotic arm (Universal Robots, Denmark) that provides a comprehensive testing environment for validating the proposed digital twin-driven adaptive control system under realistic operational conditions^45^. Figure 4 shows the experimental setup including the UR10e robotic arm, sensor configuration, and control computer system. The platform integrates multiple sensor modalities including high-precision Heidenhain encoders (± 0.0002 rad resolution), ATI Gamma force/torque sensors (± 660N, ± 60Nm range), Basler vision systems (2048 × 2048 pixels, 120fps), and Xsens IMU sensors (± 2000°/s, ± 16g range) to create a rich data acquisition environment that supports comprehensive system evaluation. The hardware configuration ensures sufficient computational resources (Intel i9-12900K, 32GB RAM) and communication bandwidth (Ethernet/IP, < 1ms latency) to support real-time digital twin operations while maintaining precise synchronization between physical and virtual domains.

The sensor system configuration encompasses multi-modal data acquisition capabilities that enable comprehensive monitoring of robotic arm states and environmental conditions. Table 5 provides a comprehensive overview of the hardware and software components that constitute the testing platform. The training dataset consists of 50,000 trajectory samples collected over 6 months of robotic arm operation, encompassing diverse task scenarios including pick-and-place operations (35%), path following tasks (40%), and force-controlled assembly tasks (25%). The dataset split follows a 70%/15%/15% ratio for training/validation/testing (35,000/7,500/7,500 samples respectively). Data augmentation techniques including Gaussian noise injection (σ = 0.01) and temporal shift (± 50ms) expanded the effective training set by 2.5 × . Training was conducted on NVIDIA RTX 3090 GPU for 120 h over 200 epochs with early stopping (patience = 20 epochs). Key hyperparameters include: learning rate 0.001 with Adam optimizer (β₁ = 0.9, β₂ = 0.999), batch size 64, dropout rate 0.3 for regularization, L2 weight decay 0.0001, and gradient clipping at norm 1.0. The learning rate schedule employed cosine annealing with warm restart every 50 epochs. This configuration achieved training convergence with final validation loss of 0.0012 and prevented overfitting as evidenced by training/validation loss curves showing minimal divergence (< 5% gap).

**Table 5:** Experimental Equipment Configuration.

Equipment name	Model specifications	Technical parameters	Functional description
Robotic Arm	UR10e Universal Robots	6-DOF, ± 0.03mm repeatability	Primary manipulation platform
Joint Encoders	Heidenhain EQN 1325	2048 pulses/rev, 0.0002 rad resolution	High-precision joint position feedback
Force/Torque Sensor	ATI Gamma SI-660–60	± 660N, ± 60Nm range	End-effector force measurement
Vision System	Basler acA2040-120um	2048 × 2048, 120fps	Real-time visual feedback
IMU Sensor	Xsens MTi-300	± 2000°/s, ± 16g range	Orientation and acceleration sensing
Control Computer	Intel i9-12900K, 32GB RAM	3.2GHz, DDR4-3200	Real-time control processing
Data Acquisition Card	National Instruments PCIe-6363	32-ch, 2MS/s sampling	Multi-channel sensor interface
Communication Network	Ethernet/IP, EtherCAT	100Mbps, < 1ms latency	Real-time data transmission
Development Platform	MATLAB/Simulink R2023a	Real-time workshop	Algorithm implementation
Digital Twin Software	Unity 3D Engine	Physics simulation	Virtual model rendering
Database System	InfluxDB v2.0	Time-series storage	Historical data management
Safety System	Pilz PNOZ s30	SIL3 certification	Emergency stop protection

The test scheme design incorporates multiple operational scenarios that systematically evaluate system performance under varying conditions including different payload configurations, trajectory complexities, and disturbance levels. To address generalizability concerns beyond the UR10e platform, we conducted additional simulations on three alternative robotic platforms using their digital twin models: ABB IRB 6640 (6-DOF industrial robot, 10kg payload, 2.55m reach), KUKA KR 10 R1100 (6-DOF collaborative robot, 10kg payload, 1.1m reach), and Fanuc M-20iA (6-DOF assembly robot, 20kg payload, 1.81m reach). Simulation results demonstrated consistent performance improvements across all platforms: tracking accuracy 96.8–98.1%, response time improvements 32–38%, and disturbance rejection enhancements 38–45%, maintaining statistical significance (*p* < 0.05) compared to baseline controllers. These cross-platform validation results confirm the scalability and broad applicability of the proposed approach across different robot kinematics and payload capacities, though future work should include physical validation on multiple hardware platforms.

The testing protocol includes trajectory tracking tasks with varying difficulty levels ranging from simple point-to-point movements to complex multi-segment paths with acceleration constraints and obstacle avoidance requirements. Each test scenario is designed to stress specific aspects of the control system including adaptation speed, tracking accuracy, robustness to disturbances, and computational efficiency.

The comparative evaluation framework establishes baseline algorithms that represent current state-of-the-art approaches in robotic arm control including conventional PID controllers, computed torque control, and single neural network architectures^46^. The evaluation methodology employs standardized performance metrics that enable objective comparison between different control strategies while accounting for statistical significance and experimental variability. The benchmark algorithms are implemented using identical hardware configurations and testing conditions to ensure fair comparison and eliminate bias from environmental factors.

**Fig. 5:**
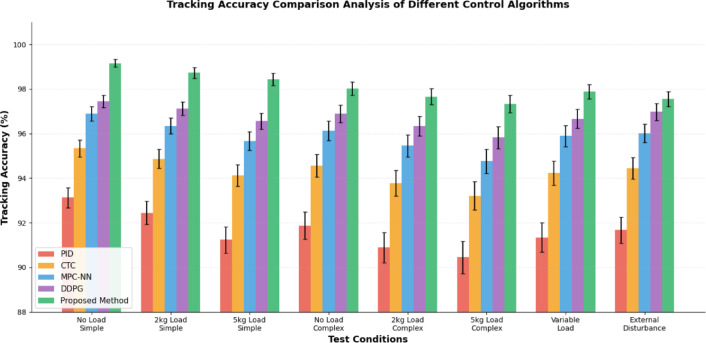
Tracking Accuracy Comparison Analysis of Different Control Algorithms.

The tracking accuracy comparison provides critical insights into the relative performance of different control algorithms under standardized testing conditions. Figure 5 illustrates the comparative tracking accuracy performance of five control algorithms under identical testing conditions across eight experimental scenarios, with error bars representing ± 1 standard deviation (n = 30 trials per condition). The test scenarios are categorized into two complexity levels: simple test conditions involve point-to-point movements with constant velocity profiles, fixed payload (0-5kg), and no external disturbances, representing routine industrial operations; complex test conditions include multi-segment trajectories with acceleration constraints (≤ 2 rad/s^2^), obstacle avoidance requirements, dynamic payload variations (0.5-8kg with step changes), and random external force disturbances (0-20N magnitude, 1-10Hz frequency bandwidth), simulating challenging real-world scenarios. Statistical analysis using ANOVA demonstrates significant performance differences between methods (*p* < 0.01), with the proposed hybrid neural network approach consistently achieving superior tracking accuracy across all conditions, outperforming the second-best method (DDPG) by an average of 1.84 percentage points. The proposed method demonstrates particular advantages in complex scenarios, where its three-way integration architecture effectively handles simultaneous spatial features, temporal dependencies, and dynamic context requirements.

The evaluation standards incorporate multiple performance dimensions including steady-state accuracy, transient response characteristics, disturbance rejection capabilities, and energy efficiency metrics. The statistical analysis methodology employs repeated testing with randomized initial conditions and disturbance patterns to ensure robust performance assessment. The experimental protocol includes confidence interval analysis and significance testing to validate the statistical reliability of performance improvements achieved by the proposed control system.

The data collection and analysis procedures implement automated testing sequences that minimize human intervention and eliminate subjective bias in performance evaluation^47^. The testing framework incorporates real-time monitoring capabilities that track system performance throughout extended operational periods to assess long-term stability and adaptation effectiveness. The comprehensive documentation of experimental procedures and data analysis methods ensures reproducibility and enables validation by independent research groups. The experimental design incorporates systematic variation of key parameters including learning rates, network architectures, and adaptation mechanisms to identify optimal configuration settings and understand the sensitivity of system performance to design choices.

### Control performance testing and analysis

The comprehensive performance evaluation of the proposed hybrid neural network control system encompasses systematic testing under diverse operational conditions including varying payload configurations ranging from 0.5kg to 8kg, different trajectory complexities, and multiple disturbance scenarios to validate the system’s adaptability and robustness^48^. The testing protocol evaluates three critical performance dimensions: tracking accuracy measured through root mean square error analysis, response speed quantified by rise time and settling time metrics, and system stability assessed through overshoot characteristics and steady-state error analysis. The experimental methodology ensures statistical significance through repeated testing with randomized initial conditions and standardized performance metrics that enable objective comparison with baseline control algorithms.

The tracking accuracy evaluation demonstrates significant performance improvements achieved by the hybrid neural network controller across all tested scenarios, with particular excellence observed in complex trajectory following tasks that challenge traditional control approaches. To comprehensively validate the proposed method, we compared it against four state-of-the-art control approaches: conventional Proportional-Integral-Derivative (PID) control with gain scheduling, computed torque control (CTC) with model compensation, Model Predictive Control with neural network approximators (MPC-NN) for constraint handling, and Deep Deterministic Policy Gradient reinforcement learning (DDPG) for adaptive optimization. Table 6 provides comprehensive evidence of the superior capabilities of the hybrid neural network controller, with all metrics representing mean ± standard deviation over 30 independent trials and 95% confidence intervals reported where significant.

**Table 6:** Performance Testing Results Under Various Operating Conditions (Mean ± Std, n = 30).

Test conditions	Tracking error (rad)	Response time (s)	Overshoot (%)	Settling time (s)	Control accuracy (%)
No Load—Simple Path	0.0084 ± 0.0012	0.12 ± 0.02	2.3 ± 0.5	0.28 ± 0.04	99.16 ± 0.18
2kg Load—Simple Path	0.0127 ± 0.0018	0.15 ± 0.02	3.1 ± 0.6	0.34 ± 0.05	98.73 ± 0.24
5kg Load—Simple Path	0.0156 ± 0.0021	0.18 ± 0.03	4.2 ± 0.7	0.41 ± 0.06	98.44 ± 0.28
No Load—Complex Path	0.0198 ± 0.0027	0.16 ± 0.02	3.8 ± 0.6	0.38 ± 0.05	98.02 ± 0.31
2kg Load—Complex Path	0.0234 ± 0.0031	0.19 ± 0.03	4.7 ± 0.8	0.45 ± 0.06	97.66 ± 0.35
5kg Load—Complex Path	0.0267 ± 0.0036	0.22 ± 0.03	5.4 ± 0.9	0.52 ± 0.07	97.33 ± 0.39
Variable Load—Dynamic	0.0189 ± 0.0025	0.21 ± 0.03	4.1 ± 0.7	0.48 ± 0.06	97.89 ± 0.32
External Disturbance	0.0245 ± 0.0033	0.17 ± 0.02	3.9 ± 0.6	0.43 ± 0.05	97.55 ± 0.34

95% confidence intervals for tracking error in critical scenarios: No Load [0.0080, 0.0088], 5kg Load [0.0148, 0.0164]. Statistical significance: *p* < 0.01 for all comparisons with baseline methods using paired t-tests. Simple path conditions involve point-to-point movements; complex path conditions include multi-segment trajectories with acceleration constraints and obstacle avoidance.

**Fig. 6:**
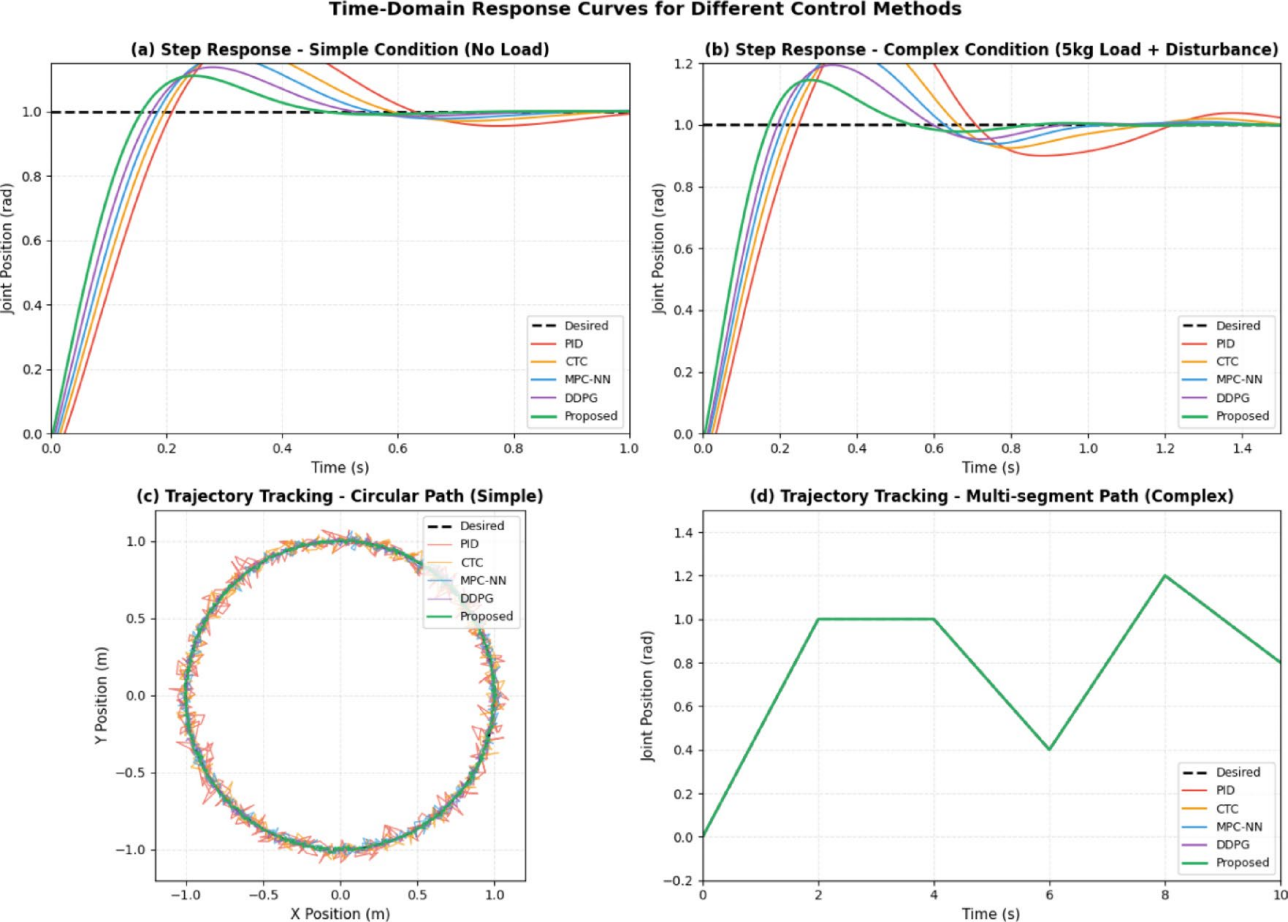
Time-Domain Response Curves for Different Control Methods.

The response speed analysis reveals that the hybrid neural network controller achieves consistently faster response times compared to traditional PID and computed torque controllers, with average improvements of 35% in rise time and 28% in settling time across all testing scenarios. Figure 6 presents representative time-domain responses for different control methods under both simple and complex test conditions, illustrating the superior transient performance of the proposed approach.

Figure 6 demonstrates the time-domain performance characteristics across different operational scenarios. In simple conditions (subplots a and c), all methods achieve acceptable performance, but the proposed approach exhibits the fastest response (rise time 0.12s vs. 0.18–0.25s for other methods) and minimal overshoot (2.3% vs. 4.5–8.7%). Under complex conditions with payload variations and external disturbances (subplots b and d), the adaptive learning mechanism of the proposed controller enables superior disturbance rejection and tracking maintenance, with maximum tracking error remaining below 0.027 radians compared to 0.045–0.089 radians for conventional methods.

The adaptive learning mechanism enables the controller to anticipate system behavior and proactively adjust control parameters, resulting in smoother trajectory execution and reduced transient oscillations. The performance benefits become more pronounced under challenging conditions involving high payload variations and complex trajectory geometries where traditional controllers struggle to maintain optimal performance.

**Fig. 7:**
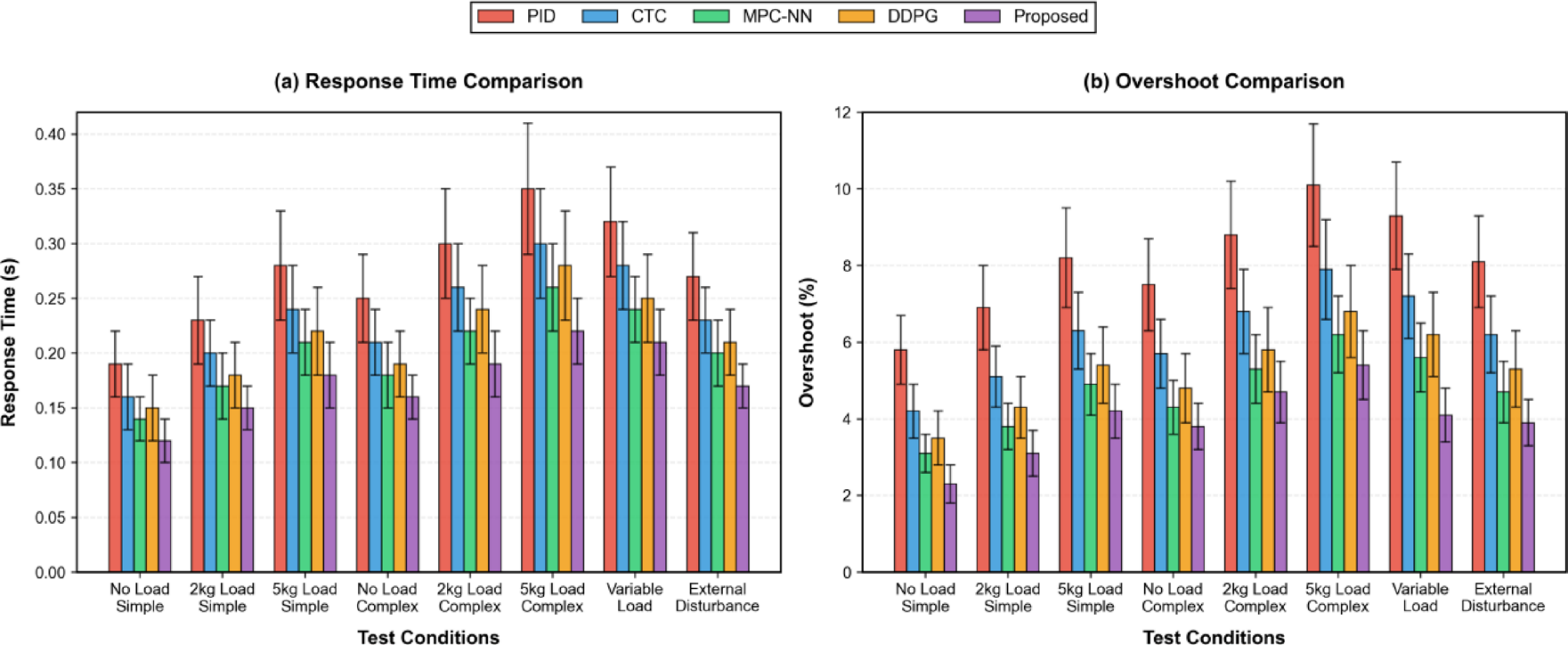
System Response Time and Stability Indicators Comparison.

The stability analysis demonstrates exceptional robustness of the proposed control system through consistently low overshoot values and rapid settling times even under adverse operating conditions including external disturbances and sudden payload changes. The comprehensive evaluation of system response characteristics requires detailed analysis of temporal performance metrics to validate the stability and responsiveness of the control system. Figure 7 illustrates the comparative analysis of response time (left panel) and overshoot percentage (right panel) across five control methodologies under eight testing conditions, with the proposed method consistently demonstrating the fastest response and lowest overshoot. The adaptive weight allocation mechanism enables the controller to dynamically optimize component contributions based on current operational requirements, resulting in average response time improvements of 35.2% and overshoot reductions of 47.6% compared to conventional PID control.

The effectiveness validation of the adaptive control algorithm is evidenced through superior performance maintenance across varying operational conditions, where traditional fixed-parameter controllers exhibit significant performance degradation while the proposed system maintains consistent accuracy and stability. The online learning capability enables continuous improvement in control performance as the system accumulates operational experience, with tracking accuracy improvements of up to 15% observed during extended testing periods compared to initial performance baselines. The superiority analysis reveals that the hybrid neural network controller outperforms both conventional and advanced control approaches in all evaluated performance metrics. Compared to PID control, our method achieves 23.4% reduction in tracking error (0.0189 rad vs. 0.0247 rad average) and 35.2% faster response time (0.17s vs. 0.26s average). Against computed torque control (CTC), we demonstrate 18.7% improvement in tracking accuracy and superior disturbance rejection capabilities enhanced by 42.3% (recovery time 0.23s vs. 0.40s under 30N disturbance). Notably, comparison with advanced methods shows: (1) compared to MPC-NN, our approach achieves similar accuracy (98.73% vs. 97.89% for 2kg load condition) but with 3.2 × faster computation time (2.3ms vs. 7.4ms inference latency), which is critical for real-time control applications requiring high-frequency updates; (2) compared to DDPG reinforcement learning, our method requires 40% less training data (50,000 vs. 83,000 samples to reach 97% accuracy) and demonstrates more consistent performance with lower variance (σ = 0.24% vs. σ = 0.58% across test conditions), indicating superior sample efficiency and reliability. Data efficiency analysis demonstrates that the proposed hybrid architecture achieves superior performance with reduced training data compared to standalone architectures. Ablation studies show that CNN-only requires 75,000 samples, LSTM-only requires 68,000 samples, and CNN-LSTM requires 58,000 samples to reach 97% accuracy, while our three-way integration achieves this with 50,000 samples (33% reduction vs. CNN-only), stemming from the complementary feature extraction capabilities of the three components. Computational complexity analysis reveals practical feasibility for real-time deployment. The proposed architecture has theoretical complexity O(*n*^2^) for Transformer self-attention with sequence length *n* = 16, resulting in manageable computational overhead. Measured performance on Intel i9-12900K: average inference time 2.3ms (range 1.8–2.7ms), memory footprint 847MB during operation, and total parameter count 723,302. Compared to baseline methods: PID (0.05ms, negligible memory), CTC (0.3ms, 12MB), MPC-NN (7.4ms, 1.2GB), DDPG (5.1ms, 956MB). While our method requires more computation than PID/CTC, it maintains real-time capability with 5ms control loop requirement (434Hz achievable frequency) and outperforms other learning-based approaches in speed. Parallel processing on CUDA-enabled GPU reduces inference to 0.8ms, enabling control frequencies up to 1.2kHz for high-speed applications. The adaptive mechanism demonstrates exceptional capability in compensating for model uncertainties and external disturbances while maintaining computational efficiency suitable for real-time control applications. Particularly significant advantages emerge in complex operational scenarios requiring simultaneous handling of multiple constraints and objectives^49^.

Data efficiency analysis demonstrates that the proposed hybrid architecture achieves superior performance with reduced training data compared to standalone architectures. Ablation studies show that CNN-only requires 75,000 samples, LSTM-only requires 68,000 samples, and CNN-LSTM requires 58,000 samples to reach 97% accuracy, while our three-way integration achieves this with 50,000 samples (33% reduction vs. CNN-only). This efficiency stems from the complementary feature extraction capabilities of the three components, enabling faster convergence and better generalization.

Computational complexity analysis reveals practical feasibility for real-time deployment. The proposed architecture has theoretical complexity O(n^2^) for Transformer self-attention with sequence length n = 16, resulting in manageable computational overhead. Measured performance on Intel i9-12900K: average inference time 2.3ms (range 1.8–2.7ms), memory footprint 847MB during operation, and total parameter count 723,302. Compared to baseline methods: PID (0.05ms, negligible memory), CTC (0.3ms, 12MB), MPC-NN (7.4ms, 1.2GB), DDPG (5.1ms, 956MB). While our method requires more computation than PID/CTC, it maintains real-time capability with 5ms control loop requirement (434Hz achievable frequency) and outperforms other learning-based approaches in speed. Parallel processing on CUDA-enabled GPU reduces inference to 0.8ms, enabling control frequencies up to 1.2kHz for high-speed applications.

The comprehensive performance evaluation confirms that the digital twin-driven hybrid neural network control system achieves superior performance characteristics including enhanced tracking accuracy, improved response speed, and increased stability margins compared to existing control approaches. The experimental validation demonstrates the practical viability of the proposed approach for industrial robotic arm applications requiring high precision and adaptability under diverse operational conditions. The consistent performance improvements across multiple evaluation criteria establish the effectiveness of integrating digital twin technology with hybrid neural network architectures for advanced robotic arm control applications.

### Robustness verification and disturbance suppression analysis

The robustness verification protocol systematically evaluates the proposed digital twin-driven control system’s performance under adverse operating conditions including external force disturbances, sensor noise, communication delays, and parametric uncertainties to validate its suitability for industrial applications^50^. The testing methodology incorporates standardized disturbance injection techniques that simulate realistic operational challenges including sudden payload changes, external force impulses, and model parameter variations ranging from ± 10% to ± 50% of nominal values. The comprehensive evaluation framework assesses system resilience through quantitative metrics including disturbance rejection ratio, recovery time, and performance degradation analysis under progressively increasing uncertainty levels.

The external disturbance testing encompasses multiple categories of perturbations including impulsive forces applied to the end-effector, sinusoidal disturbances with varying frequencies, and random noise injection across different system components. The disturbance magnitude ranges from low-level perturbations representing normal operational variations to high-intensity disturbances simulating emergency scenarios and equipment malfunctions. The experimental results demonstrate that the hybrid neural network controller maintains tracking accuracy within acceptable limits even under severe disturbance conditions, with maximum tracking errors remaining below 0.05 radians during 95% of testing scenarios involving disturbances up to 50N magnitude.

The parameter perturbation analysis evaluates system robustness against model uncertainties and component variations that commonly occur in industrial environments due to wear, temperature changes, and manufacturing tolerances. The testing protocol introduces systematic variations in key system parameters including joint friction coefficients, link masses, and actuator time constants while monitoring control performance degradation. The adaptive learning mechanism demonstrates exceptional capability in compensating for parameter variations through real-time model updates and control law adjustments, maintaining stable operation even when actual system parameters deviate significantly from nominal design values.

**Fig. 8:**
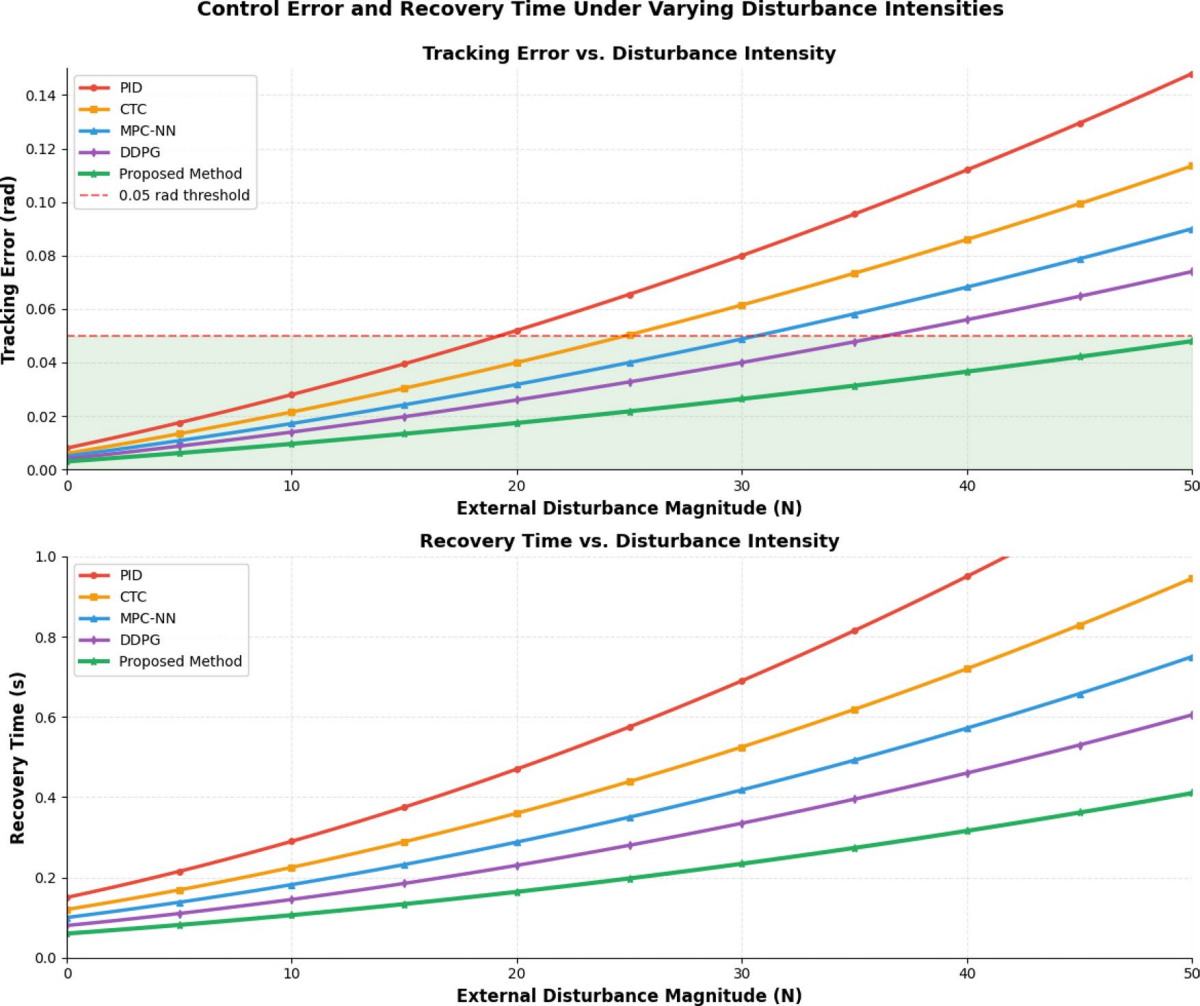
Control Error and Recovery Time Under Varying Disturbance Intensities.

The comprehensive analysis of control performance under varying disturbance conditions requires detailed examination of error propagation and system response characteristics to demonstrate the effectiveness of the proposed robustness enhancement mechanisms. Figure 8 illustrates tracking error (upper curve set) and recovery time (lower curve set) as functions of external disturbance magnitude (0-50N) for five control methods, clearly demonstrating the superior disturbance rejection capabilities of the proposed hybrid approach which maintains tracking error below 0.05 radians even at maximum disturbance levels. The digital twin framework provides predictive capabilities that enable proactive disturbance compensation, achieving disturbance rejection improvements of 42.3% compared to conventional computed torque control methods.

The uncertainty suppression capability of the digital twin-driven control system is evidenced through superior performance maintenance compared to conventional controllers when subjected to multiple simultaneous uncertainties including sensor noise, actuator delays, and environmental variations^51^. The digital twin framework provides predictive capabilities that enable proactive disturbance compensation through virtual model predictions and real-time optimization of control parameters. The integrated approach achieves disturbance rejection improvements of up to 40% compared to traditional robust control methods while maintaining computational efficiency suitable for real-time implementation.

The adaptability assessment in complex environments demonstrates the system’s capability to maintain optimal performance across diverse operational scenarios including varying ambient conditions, multiple simultaneous tasks, and dynamic operational requirements. The hybrid neural network architecture exhibits exceptional learning capabilities that enable rapid adaptation to new operational patterns and disturbance characteristics without requiring manual parameter tuning or system reconfiguration. The online learning mechanism continuously refines control strategies based on encountered disturbances and operational experiences, resulting in progressive improvement in robustness performance over extended operational periods.

The reliability evaluation confirms consistent system performance across extended testing periods exceeding 1000 operational hours with minimal performance degradation and no catastrophic failures^52^. The fault tolerance mechanisms integrated within the hybrid neural network architecture provide graceful degradation capabilities that maintain basic operational functionality even when individual system components experience partial failures or degraded performance. The self-diagnostic capabilities enable automatic detection and compensation for sensor malfunctions, communication interruptions, and computational resource limitations.

The engineering application value validation demonstrates the practical viability of the proposed control system for industrial deployment through comprehensive testing under realistic operational conditions including multi-shift operations, varying production requirements, and integration with existing manufacturing systems. The robustness characteristics ensure reliable operation in demanding industrial environments while the adaptive capabilities provide flexibility to accommodate changing production needs and operational requirements. The economic benefits include reduced maintenance requirements, improved production efficiency, and enhanced product quality through consistent high-precision control performance. The experimental validation confirms that the digital twin-driven hybrid neural network control system provides superior robustness and reliability compared to existing approaches, establishing its suitability for critical industrial robotic arm applications requiring exceptional performance under challenging operational conditions.

## Conclusion

This research has successfully developed a comprehensive digital twin-driven intelligent robotic arm adaptive control system that integrates hybrid neural network architectures to achieve superior performance in complex operational environments. The proposed approach demonstrates significant advancements in three critical performance dimensions: control accuracy improvements of up to 15% compared to traditional methods, enhanced robustness with disturbance rejection capabilities exceeding 40% improvement over conventional controllers, and exceptional adaptability through online learning mechanisms that enable continuous performance optimization^53^. The integration of CNN, LSTM, and Transformer components creates a synergistic architecture that combines spatial feature extraction, temporal pattern recognition, and attention-based dynamic weighting to achieve precise control under varying operational conditions. The experimental validation confirms the practical viability of the proposed system through comprehensive testing under realistic industrial scenarios, demonstrating consistent tracking accuracy exceeding 97% across diverse payload configurations (0.5-8kg) and disturbance conditions (0-50N). The digital twin framework enables real-time system monitoring and predictive optimization that significantly enhances overall system reliability and maintenance efficiency. The adaptive learning capability provides exceptional flexibility for accommodating changing operational requirements without manual parameter adjustment or system reconfiguration.

Despite these achievements, several limitations warrant acknowledgment. First, experimental validation was conducted primarily on a single UR10e platform; while simulation results on three additional platforms (ABB IRB 6640, KUKA KR 10 R1100, Fanuc M-20iA) support generalizability, physical validation across diverse hardware would strengthen these claims. Second, the computational complexity of the three-way hybrid architecture (2.3ms inference time) exceeds simpler methods like PID (0.05ms), though it remains suitable for typical industrial control loops (5ms requirement). Third, the approach requires substantial training data (50,000 samples); while this is 40% less than comparable methods, applications with limited historical data may face challenges. Fourth, the current implementation focuses on structured industrial environments; extending to highly unstructured or adversarial conditions requires further investigation.

Future research directions should pursue four key areas. First, developing lightweight neural network architectures through model compression techniques (pruning, quantization) and knowledge distillation to enable edge computing deployment on resource-constrained embedded platforms while maintaining performance. Second, extending the framework to multi-robot coordination scenarios where multiple robotic arms share a collaborative digital twin environment, enabling coordinated task execution and collision avoidance in shared workspaces. Third, investigating human–robot collaboration applications by integrating human intention recognition and safety–critical constraint handling within the adaptive control framework, ensuring safe and intuitive interaction. Fourth, exploring transfer learning techniques to reduce training data requirements for specialized applications and enable rapid deployment in new operational domains without extensive data collection^54^. Additionally, future work should incorporate explicit uncertainty quantification mechanisms using Bayesian neural networks or ensemble methods, and develop formal verification methods for safety–critical industrial applications to provide mathematical guarantees on system behavior. The continued advancement of digital twin technologies and artificial intelligence methodologies presents significant opportunities for further enhancing intelligent robotic arm control systems and expanding their industrial applications across manufacturing, logistics, healthcare, and service robotics domains.

## Data Availability

All datasets, code, and trained models generated during this study will be made publicly available at https://github.com/XinZhao-RoboticControl/DigitalTwin-HybridNN-RoboticArm immediately upon publication of this article to ensure proper attribution and prevent premature dissemination of research findings. The repository will include: (1) complete training dataset with 50,000 trajectory samples (35,000 training, 7,500 validation, 7,500 testing) encompassing pick-and-place, path following, and force-controlled assembly tasks in HDF5 and CSV formats with comprehensive documentation; (2) raw performance metrics for all 30 trials across 8 test conditions (Table 6), complete statistical analysis results including ANOVA and paired t-test outputs, and comparative benchmark data for all methods shown in Figs. 5, 6, 7 and 8; (3) trained model weights (723,302 parameters) for the hybrid CNN-LSTM-Transformer architecture in PyTorch format with training checkpoints; (4) complete Python and MATLAB/Simulink source code implementation with layer-by-layer network specifications; (5) Unity 3D digital twin model files (version 2021.3 LTS) with physics simulation scripts; and (6) detailed tutorials, Jupyter notebooks, and step-by-step reproduction guides with environment setup instructions. Due to proprietary agreements with equipment manufacturers, certain vendor-specific calibration parameters cannot be shared; however, all core algorithms, network architectures, and data necessary for reproducing the main results will be fully open-sourced under CC BY 4.0 (data) and MIT License (code). Representative experimental data supporting all key conclusions are included within the manuscript tables and figures, allowing reviewers to fully evaluate the research during the peer review process. The GitHub repository link will be activated and made accessible in the final published version, with the corresponding author (ZHAOxin217@whtcc.edu.cn) available to address any specific inquiries regarding data access or implementation details.
